# Supplementary material for: Transcriptomics of Long-Term Meditation Practice: Evidence for Prevention or Reversal of Stress Effects Harmful to Health
Source: Medicina (Kaunas). 2021 Mar 1;57(3):218. doi: 10.3390/medicina57030218 (PMC8001870; doi:10.3390/medicina57030218)
Supplement: Supplementary file 1 [file medicina-57-00218-s001.zip › medicina-1042090 supplementary materials/Figure S1-S3 Network 3.docx]

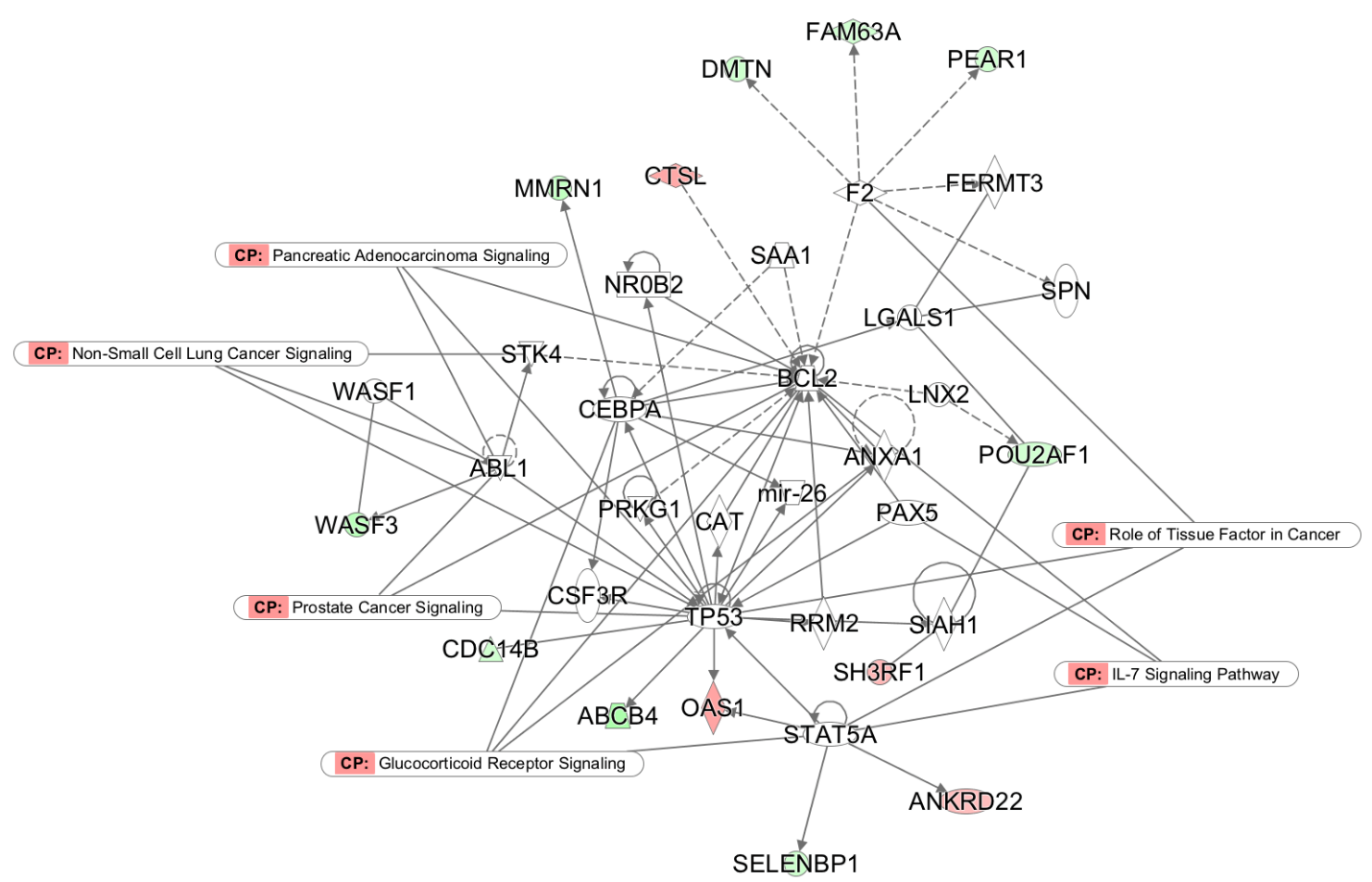


**Figure S1:** Network 3 (p-score 18): Cellular movement, immune cell trafficking, hematologic system development and function. Genes color-coded in red are upregulated, and those in green downregulated, in the TM group. CP indicates canonical pathways. TP53 (tumor protein 53, also known as p53) and BCL2 (B-cell lymphoma 2) are core molecules. TP53 is directly affected by *CDC14B* (downregulated in TM group) and in turn affects *ABCB4* (downregulated in the TM group) and *OAS1* (upregulated in the TM group). TP53 and BCL2 are connected to the role of tissue factors in cancer as well as to several cancer signaling pathways. TP53 is also indirectly connected to the glucocorticoid receptor signaling pathway through STAT5A and BCL2.


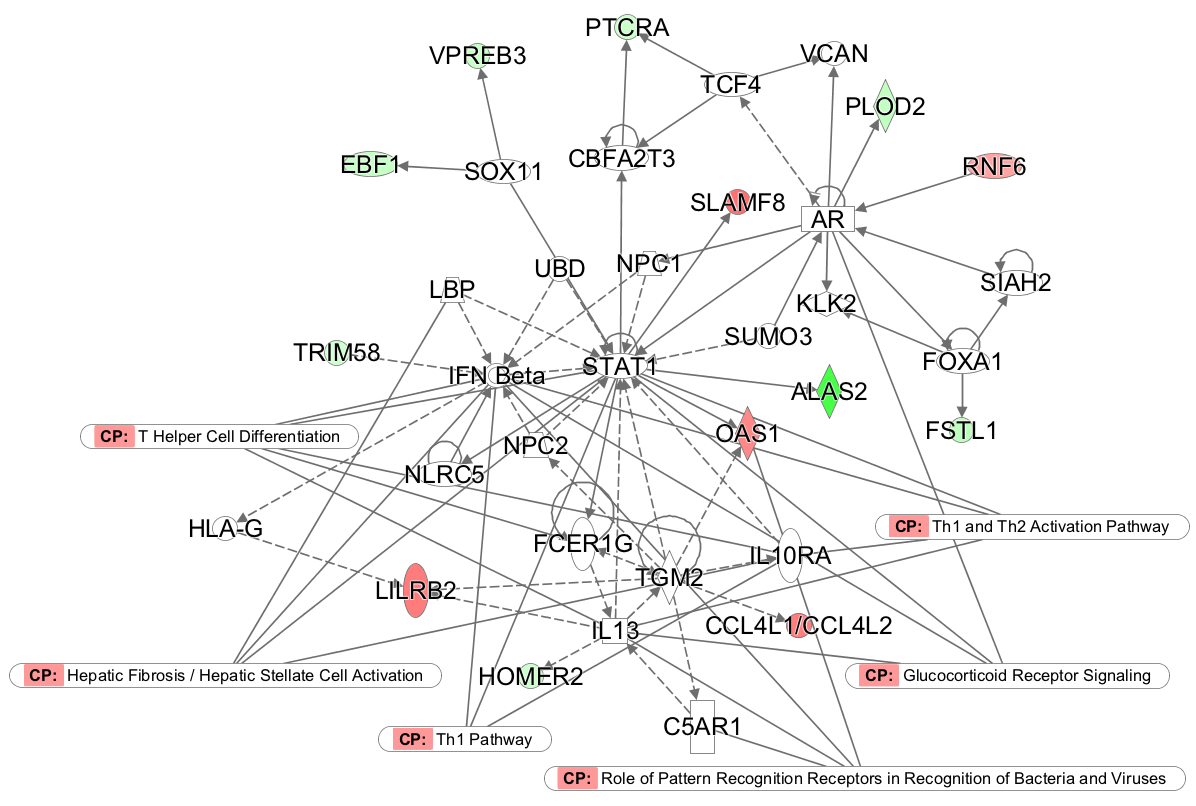


**Figure S2:** Network 4 (p-score 17): Cellular function and maintenance, hematologic system development and function, and infectious disease. Genes color-coded in red are upregulated, and those in green downregulated, in the TM group. CP indicates canonical pathways. STAT 1 (signal transducer and activator of transcription 1) is a core molecule. It is strongly affected by *ALAS2* (downregulated in the TM group) and is connected with glucocorticoid receptor signaling, T-cell helper cell differentiation, and the androgen receptor (AR). *TRIM58* (downregulated in the TM group) is connected to another core molecule, interferon beta (IFNβ), which is involved in T helper cell differentiation and in the function of pattern recognition receptors that recognize bacteria and viruses.


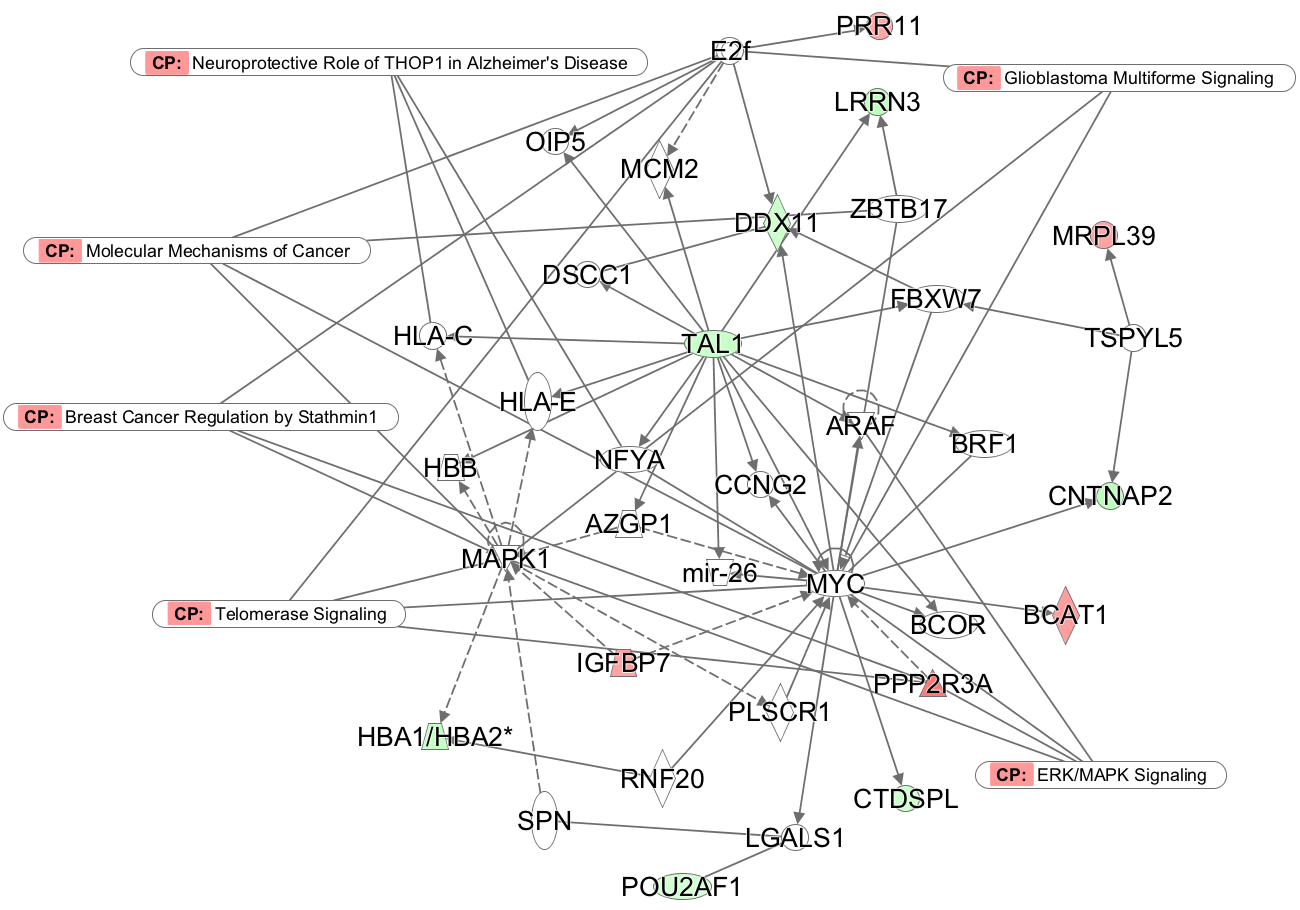


**Figure S3:** Network 5 (p-score 15): Cell cycle, cancer, organ morphology**.** Genes color-coded in red are upregulated, and those in green downregulated, in the TM group. CP indicates canonical pathways. TAL1 (T-cell acute lymphoblastic leukemia 1) is a core molecule known to connect with the telomerase signaling pathway. *TAL1* (downregulated in the TM group) directly influences the central molecule, MYC (**Myc proto-oncogene protein),** which is directly connected with telomerase signaling and with molecular mechanisms of cancer. It also directly connects with *BCAT* (upregulated in the TM group) and indirectly with *IGFBP7* (upregulated in TM) and is important in ERK/MAPK regulation of cell growth and differentiation.
