# Supplementary material for: Transcriptomics of Long-Term Meditation Practice: Evidence for Prevention or Reversal of Stress Effects Harmful to Health
Source: Medicina (Kaunas). 2021 Mar 1;57(3):218. doi: 10.3390/medicina57030218 (PMC8001870; doi:10.3390/medicina57030218)
Supplement: Supplementary file 1 [file medicina-57-00218-s001.zip › medicina-1042090 supplementary materials/Table S1 Primer Sequences for qPCR Reactions.doc]

**Table S1: Primer Sequences for qPCR Reactions. F = Forward Primer, R = Reverse Primer**

| **Target gene** | **Primer sequence (5’ 🡪 3’)** | **Product size** |
| --- | --- | --- |
| *ITGB3* | F:TCACCATCCACGACCGAAAA | 108 |
|  | R:TGAAGGTAGACGTGGCCTCT |  |
| *ITGB5* | F: AGATGTGTGAGAAGTGCCCC | 88 |
|  | R: GGTTTCCCAGAGTGGAGCAG |  |
| *CXCL10* | F: TGCAAGCCAATTTTGTCCACG | 169 |
|  | R: CTGCATCGATTTTGCTCCCC |  |
| *CDKN1C* | F: CGATCAAGAAGCTGTCCGGG | 72 |
|  | R: ACTTCTCAGGCGCTGATCTC |  |
| *TAL1* | F: TGAAGAGGAGACCTTCCCCC | 76 |
|  | R: GTGAAGATACGCCGCACAAC |  |
| *LMNA* | F: GAGGCTCTGCTGAACTCCAA | 179 |
|  | R: AGCCTGTTCTCAGCATCCAC |  |
| *SOCS3* | F: TCTGTCGGAAGACCGTCAAC | 106 |
|  | R: CCTTAAAGCGGGGCATCGTA |  |
| *IGFBP7* | F: CATCACCCAGGTCAGCAAGG | 85 |
|  | R: GGCACCAGTGACATTCCAGA |  |
| *AHSP* | F: CCTGTTAGACCTGAAGGCAGAT | 159 |
|  | R:AGTTCATCCAGTCCTCCACCA |  |
| *TLR4* | F: AAAATCCCCGACAACCTCCC | 175 |
|  | R: AGAGGTGGCTTAGGCTCTGA |  |
| *MYL9* | F: TTTGACCAGTCCCAGATCCAG | 140 |
|  | R: CCCTCCAGGTATTCGTCTGT |  |
| *ALOX12* | F: TCCAACTGCACGAGATCCAG | 134 |
|  | R:GGTGTAGCGGATATGGGGGA |  |
| *CD22* | F:GATTGGAGCCGGTGAAGGTC | 188 |
|  | R:CTTGAGCCCACAGATTGCCA |  |
| *HES4* | F:CGCTCAGCTCAAAACCCTCA | 87 |
|  | R:ATCTCCAGGATGTCCGCCTT |  |
| *HPRT** | F:GCA GAC TTT GCT TTC CTT GG | 93 |
|  | R:TAT CCA ACA CTT CGT GGG GT |  |
| *IL-1B* | F: CAGAAGTACCTGAGCTCGCC | 153 |
|  | R: AGATTCGTAGCTGGATGCCG |  |

*Reference gene
